# Spatial assortment of soil organisms supports the size-plasticity hypothesis

**DOI:** 10.1038/s43705-022-00185-6

**Published:** 2022-10-19

**Authors:** Alain Isabwe, Haifeng Yao, Shixiu Zhang, Yuji Jiang, Martin F. Breed, Xin Sun

**Affiliations:** 1 Key Laboratory of Urban Environment and Health, Ningbo Observation and Research Station, Fujian Key Laboratory of Watershed Ecology, Institute of Urban Environment, Chinese Academy of Sciences, Xiamen, 361021 China; 2 Zhejiang Key Laboratory of Urban Environmental Processes and Pollution Control, CAS Haixi Industrial Technology Innovation Center in Beilun, Ningbo, 315830 China; 3 University of Chinese Academy of Sciences, Beijing, 100049 China; 4 Key Laboratory of Mollisols Agroecology, Northeast Institute of Geography and Agroecology, Chinese Academy of Sciences, Changchun, 130102 China; 5 State Key Laboratory of Soil and Sustainable Agriculture, Institute of Soil Science, Chinese Academy of Sciences, Nanjing, 210008 China; 6 College of Science and Engineering, Flinders University, Bedford Park, SA 5042 Australia

**Keywords:** Biodiversity, Community ecology, Conservation biology, Biodiversity, Community ecology

## Abstract

The size-plasticity hypothesis posits that larger size organisms are less plastic in their metabolic rates and, therefore, are more strongly environmental-filtered than smaller organisms. Many studies have supported this hypothesis by evaluating the relative roles of environmental filtration and dispersal for different taxonomic groups of soil organisms. Most observations are made at large spatial scales, which are assumed to have a wide array of varying habitats. However, since urbanization causes habitat fragmentation at smaller regional scales, testing the size-plasticity hypothesis at this scale would help better understand the spatial assortment of urban soil organisms which, in turn, would help to develop improved management and conservation strategies for urban soil health. Here, we used DNA metabarcoding on five groups of soil biota (bacteria, fungi, protists, nematodes, and invertebrates) to assess the relative importance of dispersal and environmental filters to examine the size-plasticity hypothesis at this spatial scale in an urban environment. We observed strong distance-decay of community similarities associated with higher levels of stochastic changes in bacteria, nematode, and protist communities but not fungal or invertebrate communities. Bacterial communities occupied the widest niche followed by protists and nematodes, potentially because of their higher dispersal abilities compared to the larger soil organisms. Null deviation of communities varied with taxonomic groups where bacteria and nematodes were mainly driven by homogenizing dispersal, protists and fungi by drift, and soil invertebrates by environmental selection. We further identified a small percentage of locally-adapted taxa (2.1%) that could be focal taxa for conservation and restoration efforts by, for example, restoring their habitats and enhancing their regional connectivity. These results support the size-plasticity hypothesis at the relatively unexplored regional scale in an urbanization context, and provide new information for improving urban soil health and sustainable city models.

## Introduction

Soil biota represents a large fraction of global biodiversity and is crucial for biochemical cycling, regulation of primary productivity, biomass decomposition, and maintenance of soil health [1, 2]. Increasing evidence shows that soil biota is directly and indirectly impacted by climate change, population growth, agricultural intensification, and urbanization [1, 3–6]. These activities may lead to soil biota extinction, alteration of their trophic dynamics, and increasing spatial fragmentation of their habitats [5, 6]. It is, therefore, crucial to evaluate the spatial patterns and ecological processes that shape soil biota to help enable improved and more targeted conservation and restoration approaches [7].

Global expansion of urban areas in recent years has induced substantial changes in soil physicochemical characteristics which in turn influence soil biota. Urbanization causes land-use transitions through which fragmentation of soil habitats is inevitable [5, 8]. This fragmentation, from large continuous natural areas to small habitat patches, has strong effects on soil biodiversity [9, 10]. Among the habitat patches in urban landscapes, green spaces serve as increasingly important reservoirs of soil biodiversity as the surrounding areas are developed [11, 12]. Given that spatial patterns of soil biota should be considered when assessing the ecological conditions of urban soil biota [13], our knowledge about spatial assortment of different taxonomic groups of organisms in urban soils is crucial but poorly developed.

Soil biota encompasses both micro- and macro-organisms and is extremely variegated in terms of their morphology, biomass, diversity, and lifestyles. Their spatial patterns are generally shaped by both deterministic and stochastic processes [14–16]. These two distinct but complementary ecological processes maintain species diversity at local, regional, and global scales [17–19]. Deterministic processes are dominated by environmental filters which result from habitat preferences of species. Stochastic processes manifest mainly as random changes in species compositions [20]. Although these processes act simultaneously, their relative roles vary considerably across spatial scales [17, 18] and among different taxonomic groups [21]. Previous studies have revealed these processes for different taxonomic groups of soil biota in forests [21], farmlands [22], and in contaminated soils [23]. Evidence from large spatial scales (e.g., continental-scale) has supported the size-plasticity hypothesis which posits that smaller organisms are more plastic in their metabolic rates and, therefore, less impacted by environmental filters than larger organisms [24, 25]. According to the size-plasticity hypothesis, dispersal abilities should be reduced for the larger rather than the smaller taxonomic groups of soil biota due to the smaller organisms having greater metabolic plasticity and greater environmental tolerance. In other words, the strength of deterministic processes should increase with increasing sizes of organisms, in such a way that smaller organisms should occupy more diverse habitats than larger ones.

These expectations are also reflected in the niche breadth concept, which refers to the diversity of resources used or environments occupied by an individual, population, species, or clades [26]. Briefly, an organism with a broader niche should be more metabolically flexible at the community level. Both the niche concept and the size-plasticity hypothesis are central to estimating the risks of community collapse in a changing environment because smaller organisms have been assumed to be at greater risk of extinction as a result of higher stochastic fluctuations [27]. The two concepts, however, have rarely been integrated to detect taxa that are prone to dispersal or environmental filters. Moreover, studies investigating the relative importance of environmental filters and dispersal potential for different taxonomic groups of soil biota at regional scales are lacking. Testing this hypothesis at a regional scale is important as larger spatial scales allow for a greater diversity of organisms to coexist through ecological selection (e.g., latitudinal gradients of a taxonomic group might commonly vary by orders of magnitude between high and low latitudes), plus, from an applied perspective since conservation and restoration approaches are often implemented at regional (or smaller) scales [20, 28].

**Fig. 1:**
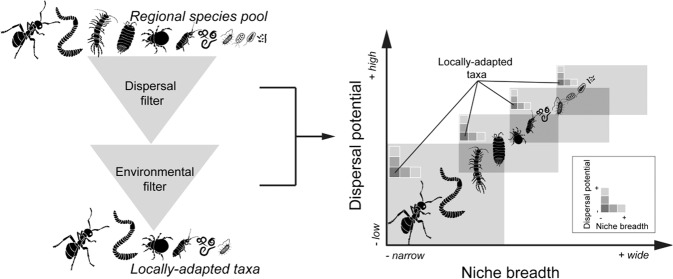
Conceptual figure of soil biota at higher taxonomic levels within which the size of soil organisms tends to increase in relation with their dispersal potentials and niche breadths. Regional species pools may be reduced into taxa that can only occur at a particular site (local communities) through dispersal and environmental filters (**left panel**). Taxa that simultaneously occupy a narrow niche breadth and exhibit low dispersal potential could be defined as the locally-adapted taxa. Dispersal abilities should be lower for the larger than the smaller organisms as a result of greater metabolic plasticity and greater environmental tolerance of the later. Smaller soil biota should occupy a wider niche breadth because they are easily dispersed (**right panel**).

Recently, Zinger et al. showed that the size of soil biota was an important determinant of community assembly, and soil mesofauna was more stochastically-assembled than microorganisms in a tropical forest of French Guiana [21]. Hypothetically, the regional species pool should be reduced into taxa that generally occupy a particular site due to dispersal and environmental filters. Smaller soil organisms should occupy a broader niche than the larger organisms because they disperse more easily (Fig. 1). Only limited studies have jointly compared assembly processes in soil micro- and macroorganisms, due in part to technical limitations in characterizing soil biota. Soil biomonitoring has greatly advanced recently via the advancement of DNA sequencing approaches, such as metabarcoding which has created an opportunity for rapid generation of soil biota data. This breakthrough has allowed detailed and comprehensive characterization of soil biota across space and time [29, 30].

In this study, we evaluated the strength of environmental filters and dispersal potential across different groups of soil biota along an urbanization gradient in a subtropical region. We hypothesized a reduced influence of dispersal and greater influence of environmental filters on the larger soil organisms compared with smaller organisms. Accordingly, we expected stochastic processes to dominate soil micro-organisms. Specifically, our study aimed at answering three questions: (1) how do environmental filters act on different taxonomic groups of soil organisms along an urbanization gradient? (2) Do soil bacteria communities exhibit stronger dispersal abilities than soil macro-organisms? (3) Is the broader niche breadth being occupied by smaller than larger soil organisms? Our results support the size-plasticity hypothesis and, *in tandem* with the niche breadth concept, yielded important insights for urban soil biota conservation and management at a time when the world’s population is more urbanized than ever before and continues to expand at an unprecedented rate [31].

## Materials and methods

### Study area and sampling design

This study was conducted in Xiamen, Fujian, China. Xiamen has a subtropical monsoon climate with an average annual temperature of 21 °C and an average annual rainfall of 1137 mm [32]. Under rapid urban development, Xiamen has experienced a decrease in farmland and an increase in built-up land by, respectively, 14.0% and 15.7% from 1980 to 2015 [33]. The sampling campaign was carried out in December 2020, the season during which optimum soil diversity is observed in the subtropics. Sampling plots were established based on the level of urbanization from fully urban areas in south east toward the mountain forests in the northwest. Briefly, four plots each in four urban, four suburban parks, and three forest sites were surveyed. The sampled urban parks were Zhongshan Park (118.09E, 24.46N), Nanhu Park (118.11E, 24.48N), Zhonglun Park (118.15E, 24.49N), and Wuyuanwan Wetland (118.17E, 24.52N). The sampled suburban parks were Jiageng Park (118.11E, 24.57N), Jingxian Park (118.11E, 24.58N), Ningbao Park (118.06E, 24.57N), and Ridong Park (118.04E, 24.57N). The sampled forest plots were located in Huweishan (118.09E, 24.49N), Xianyueshan (118.09E, 24.50N), and Tianzhusan (117.92E, 24.59N) forests (Fig. S1). Each plot in the urban, suburban parks and forest was selected based on the dominant vegetation type including grassland, grassland and shrub, grassland, trees and shrub, or tree with grassland. Three soil samples spanning a distance of ca. 100 m from each other were collected by using a soil sampler probe, thoroughly mixed to make a composite sample and then transported to the laboratory in clean polystyrene bags.

### Environmental variables

Immediately after sample collection, soil pH and moisture were measured by using a pH meter and gravimetric methods, respectively. The total organic carbon was measured using the potassium dichromate oxidation method with external heat. The concentration of total nitrogen and total carbon were quantified by using TOC/TN-VCPH elemental analyzer (Shimadzu, Kyoto, Japan). The resulting data were used to estimate the soil carbon-nitrogen ratio. Furthermore, the inductively coupled plasma mass spectrometry with ICP-MS Agilent 7500CX (Agilent Technologies, Santa Clara, CA, USA) was used to quantify the concentrations of total phosphorus and total sulfur through batch triacid digestion.

### DNA extraction and soil biota profiling

About 10 g of soil samples were thoroughly homogenized and 500 mg were sub-sampled for DNA extraction using the MP FastDNA spin kit (MP Biomedicals, Solon, OH, USA). The soil DNA was subjected to high-throughput amplicon sequencing on Illumina NovaSeq PE 250 (Illumina Inc., San Diego, CA, USA) by using a paired-end method. Four genes encoding for different soil organisms were targeted through specific primer pairs designed for PCR amplification (Table 1). The obtained paired-end sequence data were processed with QIIME2 as follows: the raw sequence data were processed using the DADA2 pipeline to filter out low quality sequences, denoising, and removing chimeras and obtain amplicon sequence variants (ASVs). Taxonomic assignments were performed by matching the representative sequence to specific annotation databases for each gene target (Table 1). To allow better comparison of distribution patterns among the studied soil organisms, the obtained ASV tables were rarefied at the same cut-off (i.e., 20 000 sequences per sample) by using the *phyloseq* package in R.

**Table 1:** Differentiating soil biota into five taxonomic groups based on the sequencing of specific gene targets and associated annotation database.

Taxonomy	Region	Primer pair [ref.]	Annotation database [ref.]
Bacteria	16S rRNA	515F/907R [78]	Greengenes [79]
Fungi	ITS	ITS1F/ITS2R [80]	Unite [81]
Protists	18S rRNA	TAReukFWD1F/TAReukREV3R [82]	PR2 [83]
Nematodes	18S rRNA	NF1/18Sr2b [84]	SILVA [85]
Invertebrates	COI	mICOI-intF/jgHCO219 [86]	COI database [87]

### Data analysis and visualization

Data analyses were carried out in R (v. 4.0) [34] and visualizations were aided by the packages *ggplot2* and *ggpubr* [35, 36]. Quantified environmental variables in the urban, suburban, and forest plots were compared using the Kruskal-Wallis rank sum tests, and were visualized in a Principal Component Analysis (PCA) ordination. Prior to ordination, the environmental variables were log (*x* + 1) transformed, with exception of the pH. The function “autoplot” of the package *ggfortify* was used to perform PCA. The Bray–Curtis similarity matrices were calculated using the *vegan* packages. These matrices were regressed against a matrix of pairwise geographical distances between the sites [37] to obtain distance-decay relationships as means to evaluate distribution patterns of the five taxonomic groups. The relationships between spatial and community matrices were assessed using Mantel tests, and the significance level was estimated after 999 permutations as per *ecodist* package [38]. The community dissimilarity matrices measured with Jaccard index were further regressed against a pairwise Euclidean distance of the sampled environmental variables to detect community–environment relationships. Then the Spearman’s correlations between the matrices and each of the measured environmental variables were calculated and visualized using *ggcor* package.

### Neutral community model

The neutral community model was used to test the importance of stochastic processes for each taxonomic group as means to test the size-plasticity hypothesis. The neutral community model assumes that stochastic processes are associated with a significant relationship between the abundance and occurrence frequency of taxa collected across different local sites in a region [39]. In practice, the neutral community model returns a parameter *Nm* which is an estimate of dispersal between communities with *N* being the metacommunity size (i.e., total number of sequences) and *m* is the migration rate. The model fit was evaluated using the proportion of local communities in which each ASV is detected, and their abundance in the metacommunity which represents regional species pool. This was done by using non-linear least-squares method with the *minpack* package to generate the best-fit distribution curve. ASVs above and below 95% confidence intervals of the neutral community model fit are assumed not to be stochastically distributed [40, 41].

### Null deviation

To improve our observations and to confirm the size-plasticity hypothesis, the neutral community model was complemented with null deviation of communities. This model uses a modified Raup–Crick dissimilarity metric that is independent of sample size and allows the calculation of the proportion of observed dissimilarities across samples that are higher than those estimated from the null model after 1000 randomizations [42]. The output results range from –1 to 1, where values approaching –1 (i.e., –0.95 to –1) indicate homogenizing dispersal, and values near 1 (0.95–1) indicate environmental selection, and the values –0.95 to 0.95 indicate drift.

### Niche breadth

Through the niche breadth concept, we expected that a taxonomic group with a wider niche breadth to be more metabolically flexible [43]. The niche breadth was calculated by using Levins’ niche breadth index (*B*):

$$Bj = 1/\mathop {\sum }\limits_{j = 1}^N P_{{{{{{{{\mathrm{ij}}}}}}}}}^2$$

where *Bj* is the habitat niche breadth of an ASV *j* in the whole species pool; *N* is the total number of communities of each metacommunity; *Pij* is the proportion of an ASV *j* in community *i* [43]. In this model, high *B* indicates that an ASV occurs widely and evenly along a wide range of sampling sites resulting in the wider niche breadth. The average *B*-values from all taxa in a single community as an indicator of habitat niche breadth at the community level. Whereas habitat specialists should have low *B*-values, habitat generalists should have higher *B*-values and be more evenly distributed along a wider range of habitats compared with habitat specialists. We ran this analysis using functions available in the *EcoUtil* package [44].

### Identifying locally-adapted taxa

In this study, locally-adapted taxa were defined as taxa that emerge from the regional species pool to become suitable to only a habitat type (Fig. 1). We assumed that each of the studied soil organisms (ASV level) underwent two major ecological filters before being observed at a given ecosystem type. First, some taxa are dispersal-filtered. These taxa are expected to be above or below the neutral community model as they may not fit the model distribution due to low dispersal abilities. Second, among taxa that are found above and below the model predictions, some taxa may be environmental-filtered. Hypothetically, these taxa may reach a given locality but are unable to adapt to local environmental conditions (e.g., under selective pressure of soil moisture, pH etc.). Through the niche breadth, these taxa are the specialists because of their abilities to cope with habitat-specific environmental condition. They have significantly low *B*-values compared to expected patterns under 999 randomizations. To improve the accuracy of these taxa, we selected the locally-adapted taxa at the intersection between the specialist taxa and taxa above or below the neutral community model predictions. The responses of the locally-adapted taxa to environmental changes were assessed by means of redundancy analysis (RDA).

## Results

### Environmental overview

The concentrations of total sulfur and total phosphorus were significantly higher (*p* < 0.05) in the urban than suburban parks and forest sites (Fig. S2). Soil pH was significantly lower (*p* < 0.05) in forests than the urban and suburban parks, and there was no clear separation between the suburban and urban sampling sites when all samples were pooled in a PCA ordination (Fig. S3). Other environmental variables such as moisture, total nitrogen, total organic carbon, and total carbon did not vary significantly among the three ecosystem types (*p* > 0.05 in each case).

### Stronger distance-decay of community similarity for soil bacteria than other soil biotas

**Fig. 2:**
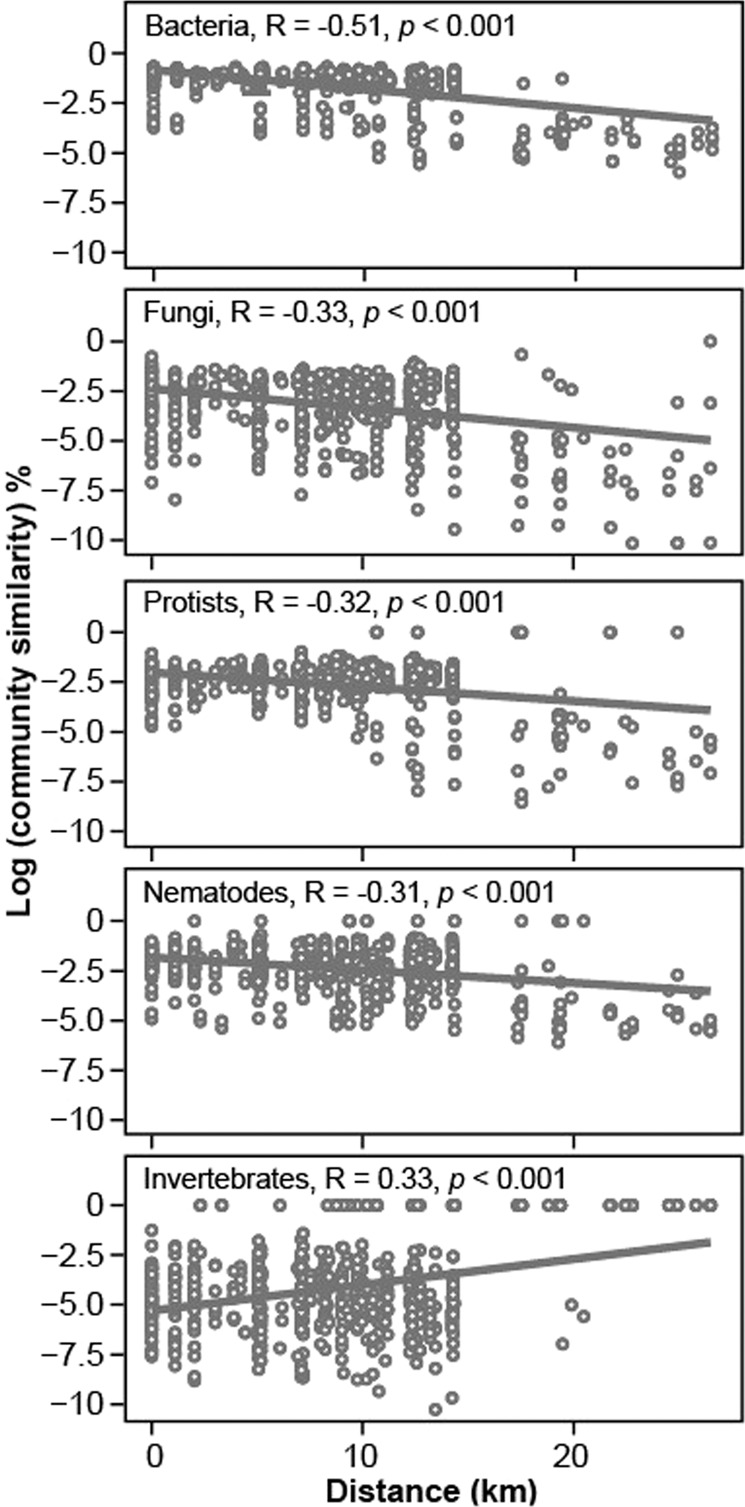
Distance-decay relationships (distance–decay relationships) of soil biota in a subtropical urban region of Xiamen, China. The Mantel statistic R and the significance levels (*p*) are presented. Significant negative Mantel’s *r* values indicate decrease of similarity between any two sites as the distance between them increases. Bray–Curtis similarity matrices were calculated using rarefied ASVs tables, log-transformed, and plotted against the geographical distance.

The rarefied communities (Fig. S4) were used for further analyses including analysis of spatial change of communities through distance-decay relationships supplemented with Mantel tests. This analysis revealed clear patterns of different taxonomic groups of soil biota (Fig. 2). Distance-decay relationships were stronger for bacteria (*r* = −0.51; *p* < 0.001) than for fungi (*r* = −0.33, *p* < 0.001). Similar level of soil community distance decay was observed for nematodes (*r* = −0.32, *p* < 0.001) and protists (*r* = −0.31, *p* < 0.05). Compared with other groups, soil invertebrates exhibited an opposite trend of distance-decay relationships (*r* = 0.33, *p* < 0.01). The comparison of community similarity matrices of the five taxonomic groups showed that the similarities were significantly different among the groups (*p* < 0.001) except between protists and nematodes (Fig. S5). Despite the low number of ASVs, both protists and nematodes had higher community similarity than fungi and invertebrates. The lowest community similarity was observed for invertebrates within the studied region that spans ca. 27 km between the most distant sites.

### High levels of stochastic change in bacteria and nematodes but not fungi, protists, or invertebrates

**Fig. 3:**
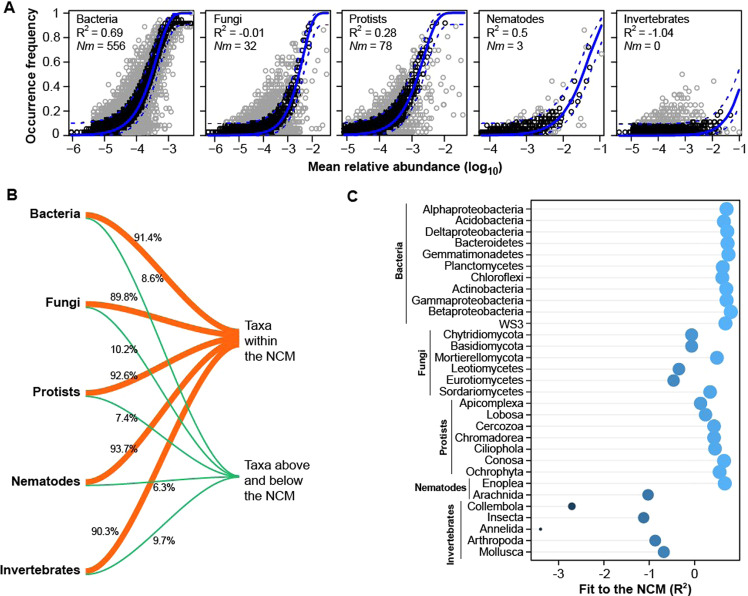
Neutral community model fit of five taxonomic groups of soil biota along an urbanization gradient in Xiamen, China. **A** The R^2^ reflects the strength of relationship between the occurrence frequencies and the relative abundance of, from left to right, bacteria, fungi, protists, nematodes, and invertebrates. The solid line indicates the fitted model for the neutral community model and the dotted lines represent 95% confidence intervals above and below the model prediction. The *Nm* is the product of the metacommunity size (*N*) and the migration rate (*m*) indicating dispersal potential among the studied size-fractioned soil biota. **B** The percentages of taxa that perfectly fitted the neutral community model, and taxa observed above and below the model prediction for each taxonomic group. **C** Class-level fit-to-the model for the different phyla of the studied taxonomic groups.

The neutral community model was used to assess the effect of stochastic processes on the assembly of each group of soil biota. Stochastic processes accounted for 69% of the variation in bacterial community assembly and 50% for nematodes. Like protists (28%), the role of stochastic processes in structuring fungi (<1%) and invertebrates (<1%) was low and not significant (Fig. 3A). The parameter *Nm* determines the relationship between the occurrence frequency, the size of the regional species pool and the level of migration rate was greatest for bacteria (*Nm* = 556), followed by protists (*Nm* = 82), fungi (*Nm* = 32), and nematodes (*Nm* = 3) (Fig. 3A). Only small percentages of taxa (6–10%) were observed above and below the neutral model prediction (95% confidence intervals) as compared to the whole metacommunity size for all taxonomic groups (Fig. 3B). Specifically, there was a greater percentage of taxa that occurred more or less frequently than predicted by neutral community model for fungi (10%), followed by invertebrates (9.7%), bacteria (8.6%), protists (7.4%), and nematodes (6.3%; Fig. 3B). At the lower taxonomic levels, the neutral community model fit generally decreased in order of taxonomic classes of bacteria, nematodes, protists, fungi and invertebrates (Fig. 3C).

### Environmental filtration acts more strongly on soil macro-organisms than microorganisms

**Fig. 4:**
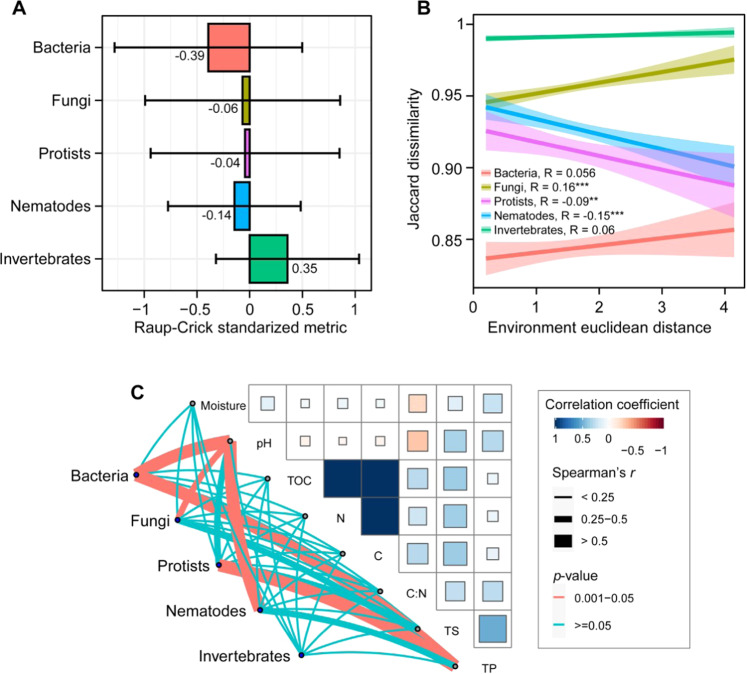
The results of standardized Raup-Crick metric of beta diversity of different taxonomic groups of soil biota and their relationships with environmental gradient. **A** Positive and negative values indicate higher and lower deviations from the null model, respectively. The values near −1 indicate homogenizing dispersal, the values between −0.95 and 0.95 indicate drift and values near 1 indicate environmental selection. **B** Linear relationships between beta diversity of the studied taxonomic groups with the Euclidean distance of environmental variables. **C** Correlation between beta diversity of different taxonomic categories with environmental variables.

By partitioning the effects of selection, dispersal, and drift using null deviation of communities for different taxonomic groups, we found that the average deviation was −0.39 and −0.14 for bacteria and nematodes, respectively (Fig. 4A). While fungal and protist communities had almost similar null deviation of −0.06 and −0.04, respectively, invertebrates had a positive mean null deviation of 0.35. The relationships between the Jaccard dissimilarity matrices of the studied soil biota with the overall environmental gradient was significant for fungi, protists, and nematodes and not for bacteria and invertebrates (Fig. 4B). The correlation analysis of soil biota beta diversity with the sampled environmental variables indicated strong positive associations between bacterial turnover and pH as well as bacteria and total sulfur. Spatial turnover in both fungi and nematodes was also strongly correlated with pH. Whereas protists were significantly correlated with total phosphorus, spatial turnover in invertebrate communities showed no significant association with environmental variables (Fig. 4C).

### Soil bacteria occupied a wider niche than other soil biotas

**Fig. 5:**
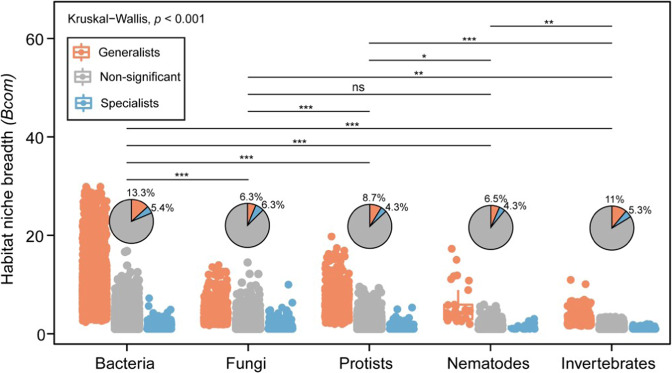
Habitat niche breadths (*Bcom*) of the studied taxonomic groups and corresponding percentages of habit specialists and generalists. The boxplots and the pie charts illustrate, respectively, the niche breadth and the percentage contribution of the specialist and generalist taxa within each group. Statistical significance among all groups and between each group pair was tested by using, respectively, the Kruskal-Wallis rank sum and Wilcoxon tests. *, *p* < 0.05, **, *p* < 0.01, ***, *p* < 0.00, ns: not-significant.

The niche occupancy (*Bcom*) of soil biota was significantly different among the studied taxonomic groups, except for fungi and nematodes (Fig. 5). The bacterial community occupied the widest niche, followed by protists, nematodes, and fungi. Although invertebrates occupied the narrowest niche, the largest number of specialist taxa (6.3%) was found for fungi (Fig. 5).

### Locally-adapted taxa reflect habitat-specific environmental conditions

**Fig. 6:**
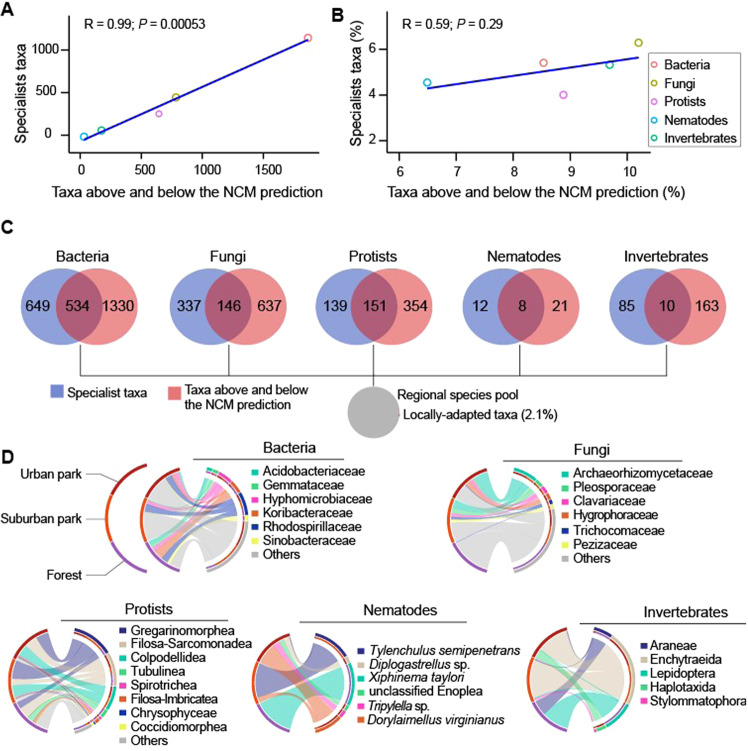
Locally-adapted taxa resulting from the intersection between specialist taxa and taxa above and below the neutral model prediction. The linear regression between the absolute number of specialists and taxa above and below the neutral community model (**A**), the percentage (**B**), intersection of the neutral community model observations and niche breadth (**C**) and taxonomic composition of the identified locally-adapted taxa (**D**).

Taxa that were adapted to a given ecosystem type (i.e., urban park, sub-urban park, and forest) were identified from the whole regional species pool by determining the taxa at the intersection between specialist taxa, and the taxa above and below the neutral community model. We observed a strong and significant relationship between the number of taxa identified as specialists and taxa that were above and below the neutral model prediction (*R* = 0.99, *p* < 0.001; Fig. 6A) when considering the absolute number of ASVs. However, this relationship was not significant when using the percentage of taxa for each group (*R* = 0.59, *p* > 0.05; Fig. 6B). The intersection of these taxa returned only 2.1 % of taxa that were locally adapted (Fig. 6C). Of these taxa, a total of 534 bacterial ASVs and 146 fungal ASVs were locally-adapted microbial taxa in the study area. There were 151 protist, 8 nematode, and 10 invertebrate locally-adapted ASVs (Fig. 6C). Although bacterial taxa were highly variable at the family level (i.e., more ASVs classified as “others”) the families *Rhodospirillaceae* and *Hypomicrobiaceae* dominated the urban parks and forest sites. Whereas the family *Streptosporangiaceae* prevailed in sub-urban parks, the family *Koribacteraceae* was the main locally-adapted taxon in forest sites. Among the dominant fungal families was the family *Archaeorhizomycetaceae* in both urban and suburban parks. The protist families *Gregarinomorphea* and *Colpodellidea* primarily dominated the urban and suburban sites (Fig. 6D). The family *Tubulinea* tended to be locally-adapted in forest sites. Among the eight locally adapted nematode ASVs, *Xiphinema taylori* was specific to forest sites while *Tylenchulus semipenetrans* and *Dolylaimellus virginianus* were dominant in sub-urban and urban parks, respectively (Fig. 6D). Finally, the locally-adapted taxa in the invertebrate group were composed of the order *Lepidoptera* and *Aranea* which were also dominant in forest sites. In contrast, the *Enchytraeida* appeared more dominant in the sub-urban and urban park sites. The later ecosystem type was uniquely characterized by ASVs belonging to the order *Haplotaxida* (Fig. 6D). Redundancy analysis showed environmental variables associated with the locally-adapted taxa (Fig. S6). Most bacterial families tended to follow an opposite direction with the environmental variables in the RDA ordination. *Streptosporangiaceae* was associated with pH, *Koribacteraceae* with moisture and *Rhodospirillaceae* with total sulfur. The fungal family *Archaeorhizomycetaceae*, the protistan *Colpodellidea*, the nematode *Dolylaimellus virginianus* and the invertebrate *Aranae* were negatively associated with increase in total carbon, total organic carbon and total nitrogen (Fig. S6).

## Discussion

We evaluated the spatial patterns of soil organisms from five taxonomic groups along an urbanization gradient in Xiamen, a subtropical region of China. We show that: (i) the soil bacterial communities exhibited stronger dispersal patterns than other soil organisms; (ii) there was a stronger environmental filtration effect on the larger (e.g., invertebrates) than the smaller soil organisms (e.g., bacteria); and (iii) the bacteria occupied a wider niche than other soil organisms. Overall, microorganisms and small soil organisms (i.e., bacteria, fungi, nematodes and protists) were generally more affected by dispersal-driven processes than large organisms (e.g., invertebrates).

### Weaker effects of environmental filters on smaller than larger soil organisms: supporting evidence of the size-plasticity hypothesis

Our results support the size-plasticity hypothesis where smaller organisms are filtered less by their environment than larger ones. With the size-plasticity hypothesis, environmental filters generally dictate how species without certain physiological traits may not occur in local communities [22, 45]. In soil systems, size-plasticity has been supported in microbial systems targeting bacteria, archaea, and fungi [22]. Both smaller and larger soil organisms should exhibit widely different degrees of metabolic flexibility, dispersal environmental tolerance, and evolutionary adaptation [21]. These features may enable some taxa to occupy wider niches, resulting in greater coexistence of species within metacommunities [46, 47].

In order to identify the relative roles of dispersal and environmental filtration-related processes that generated the observed distribution patterns, we integrated the neutral community model, the null deviation, and the niche breadth. We show that stochastic processes were dominant for bacteria and nematodes as a significant percentage of taxa (69% and 50%) could be explained by the neutral community model. Fungi, protists, and soil invertebrates were potentially structured by non-random deterministic processes. Deviation from the null model expectations in community turnover of the studied soil biota returned mean values that were significantly lower than expected by the null model for bacteria, nematodes, protists, and fungi but greater than expected for invertebrates. These results indicate that bacteria and nematodes exhibited, on average, a negative null deviation that tended to be closer to −0.95 suggesting that homogenizing dispersal has a major role in structuring both bacteria and nematodes [42]. In conjunction with their large percentage of community variation for both bacteria and nematodes, these findings explained the observed strong distance-decay patterns. The communities of fungi and protists exhibited similar patterns and underlying processes, as the null deviations were closer to −0.95 to 0.95—a range that indicates a major role of drift [42]. Invertebrates were more driven by environmental selection because the null deviation tended to be between 0.95 and 1. From these observations, the strength of environmental selection generally decreases with soil organisms’ sizes, from microbial communities to invertebrates. High dispersal rates of microbial communities could partially explain steeper distance-decay relationships observed for both bacteria and nematodes [48]. Similar patterns and processes between bacteria and nematodes could also result from the roles of nematodes in stimulating bacterial dynamics in a spatially-dependent manner [49].

Both fungi and protists underwent similar assembly processes as the neutral community model did not explain a significant proportion of taxa and null deviation, indicating the major role of drift. According to both the neutral community model observations and null deviation, niche-based deterministic processes were the main force in shaping invertebrates. This observation could be explained by the fact that, on one hand, soil bacteria have higher environmental tolerance, and rapid growth rates compared to large organisms such as invertebrates. On the other hand, both low sinking rates and high population sizes may allow them to undergo strong passive dispersal. The contrasting patterns observed for fungi, may be explained by the fact that most of their thallus comprises vegetative spreading mycelium in soil, reaching up to 100 m in some cases [50]. Therefore, in certain fungal groups, our sampling might have captured the same individual multiple times, which is less likely to occur in other taxonomic groups. Through the niche breadth concept, it is possible to identify a set of taxa that tolerate resources available in an ecosystem [26]. However, most previous studies evaluated the niche breadth across large spatial scale (e.g., many degrees of latitude), yet at these scales, climatic gradients are likely to exert an influence on resource availability [51]. Whereas the niche breadth can be defined along any number of ecological axes [52], limited number of studies have applied this concept at a regional scale influenced by human activities such as urbanization. In our study, the order of niche breadth occupancy, from the wider to narrow niche was as follows: bacteria > protists > fungi > nematodes > invertebrates. Dispersal is a major concept that explains distribution patterns, in which species distributions are explained by differences in arrival [46, 47, 53]. The ability of taxa to tolerate environmental conditions would be better assessed once the arrival of taxa (through dispersal) has been well established [47].

### Distribution patterns of soil organisms

Distance–decay relationships are widely used to test whether communities that exist closer in space are compositionally more similar than those further apart [37, 54]. Generally, communities with lower dispersal potential should have steeper trends of community similarity over a distance compared to communities with high dispersal potentials [54]. By using distance-decay relationships, many previous studies have shown distribution patterns of different taxonomic groups by gauging their spatial community turnover [e.g., 55–57], but rarely has a wide range of taxonomic groups in soils been jointly compared, as we did in this study. Fungi exhibited stronger distance–decay relationships than protists, bacteria, and soil animals in the alpine grasslands on the Tibetan Plateau [58] and in paddy soils along a transect across East Asia [18]. In previous studies, fungal communities exhibited stronger distance–decay relationships than bacteria in far-distant termite mounds of Australia [59], in dryland habits of northwest China [57], across Eastern China forests [56] and in north China agricultural soils [55]. These observations were made at large spatial scales yet biological processes act at different spatial and temporal scales [60, 61], including those that are escalated by increasing urbanization worldwide [5, 6]. In contrast to the above studies, we observed stronger distance–decay relationships for bacteria than fungi and protists at a regional scale, pointing to a non-ubiquitous distribution of soil microorganisms. The results also suggested that major taxonomic groups, within which body size traits are conserved, is a key factor influencing community assembly even at a regional scale [18, 62]. We expected to find similar distribution patterns between bacteria and fungi as microorganisms, but, while distance–decay relationships of fungi were negligible, bacteria followed similar distance–decay relationship patterns as nematodes and protists. This indicates that distribution patterns observable through distance–decay relationships may result from the presence of major dispersal barriers, dispersal limitation, or a decreasing similarity in environmental features [54]. Thus, we could go one step further evaluating the ecological mechanisms underlying the observed distribution patterns (see below).

### Locally-adapted taxa across ecosystem types

By integrating the neutral community model and niche breadth analysis, we identified a small percentage of taxa (2.1%) that appear to be locally adapted to each of the three studied ecosystem types (i.e., urban, suburban, and forest) along our urbanization gradient. These taxa could reflect environmental changes and could be of great importance in designing appropriate conservation and restoration practices in the face of increasing urban-induced spatial fragmentation.

Locally-adapted taxa from each of the five studied taxonomic groups were identified and their potential to locally colonize each of the three ecosystem types including urban, suburban parks, and forest sites. For bacteria, members of the family *Koribacteraceae* were unique to forest sites. This family plays a pivotal role in plant-biomass degradation [63] and was previously found to dominate in soils after a native forest was turned into teak plantation [64]. Although they may occur naturally in forest soils [65], their dominance in managed forests such as teak could be linked to their opportunistic behavior to high levels of available organic matter from forest debris. The purple photosynthetic bacteria (*Rhodospirillaceae* and *Hypomicrobiaceae*) that are often found in stagnant waters and mud, were among the main locally-adapted bacteria in urban parks. These taxa deserve further attention due to their extremely varying morphological and physiological features [66]. The urban and suburban parks were also enriched for locally adapted putative saprophyte fungi belonging to the family *Archaeorhizomycetaceae* that were previously found dominant in areas with greater water levels such as bald cypress-dominated swamps in USA [67] and balsam fir-dominated parks in Quebec, Canada [68]. The families *Gregarinomorphea* and *Colpodellidea* which consist mainly of the apicomplexan parasites that use invertebrates as hosts [69], co-occurred with invertebrate worms of the family *Enchytraeidae* and the family *Haplotaxida* in the urban and suburban parks. Remarkably, some members of the family *Gregarinomorphea* were previously found surviving the gut of *Enchytraeidae* [70] indicating that further research on co-occurrence of invertebrates and parasitic protists in urban soils is needed. Whereas the plant parasite nematode *Tylenchulus semipenetrans* appeared to be adapted to suburban park soils, the omnivorous nematode *Dorylaimellus virginianus* was a potential locally-adapted taxon in urban park soils, which suggests that urbanization alters the functional composition of the soil nematode community [71]. While the former tends to follow a gradient of total phosphorus increase, the later tends to follow a negative trend of total phosphorus increase. Similar to this study in which families *Araneae* and *Lepidoptera* were locally adapted in forests sites, they also comprised the major proportion of the total abundance (78%) of all invertebrates found in forests of the Azores archipelago [72]. However, both taxa differed in the way they correlated with soil moisture. Whereas *Araneae* were previously negatively correlated with soil moisture [73], *Lepidoptera* showed the opposite trend [74]. Further field and experimental studies could be useful to confirm the observed concordant negative responses of *Aranae*, *Archaeorhizomycetaceae*, *Colpodellidea* and *Dolylaimellus virginianus* taxa to organic matter availability. Noticeably, some soil pathogens were more locally-adapted in human-dominated than natural sites. For example, both the apicomplexan parasites of the family *Gregarinomorphea* (protist), and some members of the family *Pleosporaceae* (fungi), which were previously found in green spaces around Bournemouth city (UK) [75], dominated in urban and suburban sites. This calls for a deeper spatial assortment of pathogens in urban soils to help protecting plants, animals and humans.

### Implications for soil biota conservation in urban landscapes

Urbanization causes habitat fragmentation, resulting in urban green spaces being important soil biodiversity reservoirs. Due to the rapid rate of global urbanization, it is imperative for biodiversity conservation to better understand how ecological processes diverge in these urban systems [19]. In turn, understanding ecological processes that shape distribution patterns of soil organisms along environmental gradients, such as those induced by urbanization, is an important step toward conservation of soil biodiversity [17, 18]. Green spaces (e.g., urban parks, suburban parks, and natural forests) may be useful to examine the effect of urbanization on ecological processes of soil biodiversity. Communities whose distribution patterns are more strongly influenced by environmental filtering processes are assumed to be unique to a given locality [76]. Thus, to better inform management actions, restoration efforts could focus on improving regional patterns in the communities concerned. Because the movement of soil taxa is ubiquitous and facilitates important ecological mechanisms that drive local community and regional species pool composition, and hence biodiversity [77], restoring the local conditions or enhancing regional connectivity could be pathways for soil biota conservation in the face of increasing urbanization. With increasing anthropogenic threats to soil biodiversity, the potential of community assembly inferences to promote soil biota conservation should be further explored.

## Conclusion

We aimed to determine whether microorganisms exhibit stronger dispersal abilities than large soil biota along an urbanization gradient. The relevance of the size plasticity hypothesis which is generally portrayed under the above assumption was supported by our findings. One potential implication of our study is the possibility that conservation efforts could use higher taxonomic groups and inferences from community assembly to determine taxa that are locally adapted in urban soil systems, which could be useful for targeted conservation efforts. Further research is needed to determine the degree to which the size-plasticity hypothesis stands in multiple urban soils with differing magnitudes of environmental gradient, and incorporate more taxonomic groups (e.g., viruses and archaea) by taking advantage of soil DNA metabarcoding potential.

## Supplementary information

Supplementary Material

## Data Availability

Sequence data that support the findings of this study are available under BioProject ID PRJNA884290 from the NCBI Sequence Read Archives, R scripts for data analyses are provided in the supplementary materials.
