# Supplementary material for: Spatial assortment of soil organisms supports the size-plasticity hypothesis
Source: ISME Commun. 2022 Oct 19;2:102. doi: 10.1038/s43705-022-00185-6 (PMC9723746; doi:10.1038/s43705-022-00185-6)
Supplement: Supplementary file 1 — Supplementary Material [file 43705_2022_185_MOESM1_ESM.docx]

**SUPPLEMENTARY MATERIAL OF THE ARTICLE:**

**Spatial assortment of soil organisms supports the size-plasticity hypothesis**

Alain Isabwe^a,b,1^, Haifeng Yao^a,c,d,1^, Shixiu Zhang^d^, Yuji Jiang^e^, Martin F. Breed^f^, Xin Sun^a,b,c*^

*^a^Key Laboratory of Urban Environment and Health, Ningbo Observation and Research Station, Fujian Key Laboratory of Watershed Ecology, Institute of Urban Environment, Chinese Academy of Sciences, Xiamen 361021, Peoples R China*

*^b^Zhejiang Key Laboratory of Urban Environmental Processes and Pollution Control, CAS Haixi Industrial Technology Innovation Center in Beilun, Ningbo 315830, Peoples R China*

*^c^University of Chinese Academy of Sciences, Beijing 100049, Peoples R China*

*^d^Key Laboratory of Mollisols Agroecology, Northeast Institute of Geography and Agroecology, Chinese Academy of Sciences, Changchun 130102, Peoples R China*

*^e^State Key Laboratory of Soil and Sustainable Agriculture, Institute of Soil Science, Chinese Academy of Sciences, Nanjing 210008, Peoples R China*

*^f^College of Science and Engineering, Flinders University, Bedford Park, SA 5042, Australia*

***^*^****Corresponding author:* [*xsun@iue.ac.cn*](mailto:xsun@iue.ac.cn)

^1^These authors contributed equally to this paper.

**THIS SUPPLENTARY MATERIAL CONTAINS:**

- Fig. S1: Sampling map.
- Fig. S2: Comparison of the environmental variables across forest sites, suburban and urban park sites.
- Fig. S3: Principal component analysis (PCA) of the sampled environmental variables.
- Fig. S4: Rarefaction curves.
- Fig. S5: Comparison of the community similarity among different body size-fractioned categories.
- Fig. S6: Redundancy analysis of the locally-adapted taxa.
- R SCRIPTS

**Supplementary figures**


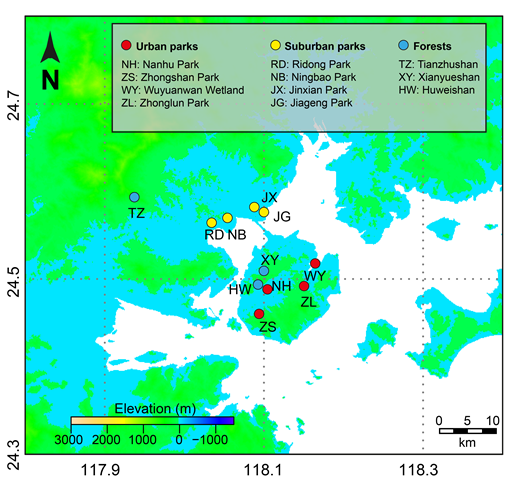


**Fig. S1** Sampling map showing the distribution of the sample points. The points are colour-coded by their corresponding ecosystem type, and the labels are the abbreviated names of the sampled sites.  The map was created using rasta package in R. The background image uses Digital Elevation Model (DEM) in GADM data freely available at <https://gadm.org/data.html>.


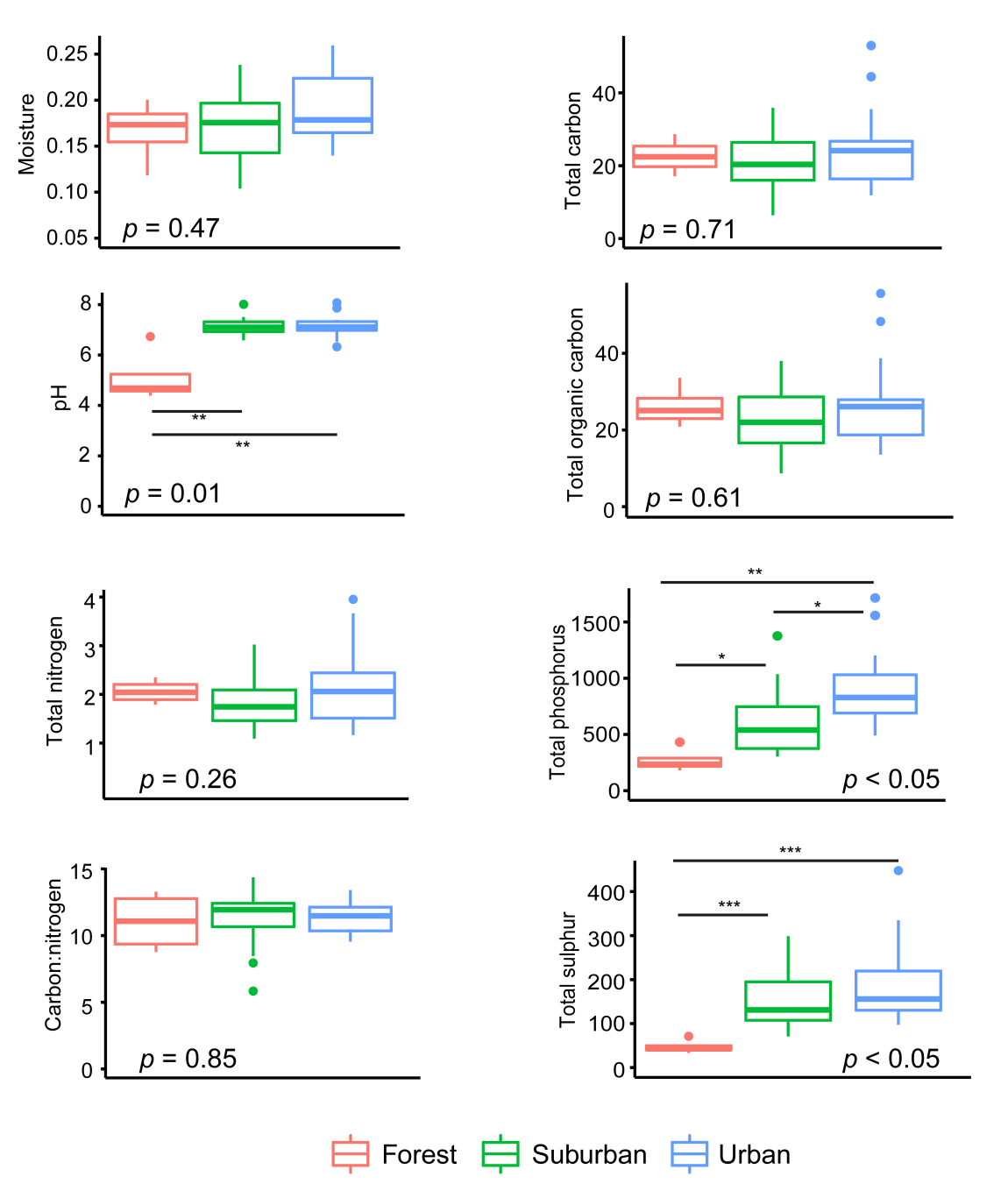


**Fig. S2** Comparison of the environmental variables across forest sites, suburban and urban park sites. Statistical significance among all groups and between each group pair were tested using, respectively, the Kruskal-Wallis rank sum and Wilcoxon tests. Whereas the *p*-values resulting from all groups comparisons, the significance levels between each group pair were not displated unless if *p* < 0.05(*), *p* < 0.01 (**) and *p* < 0.001 (***).


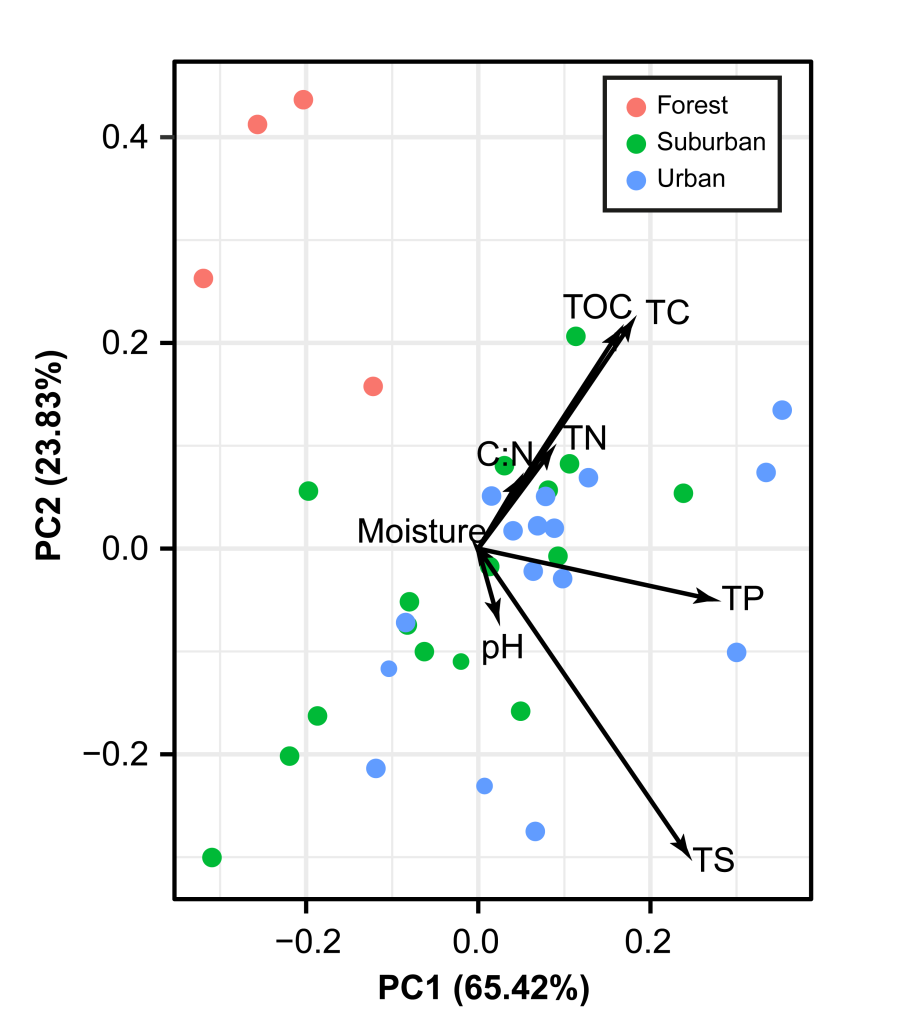


**Fig. S3** Principal Component Analysis (PCA) of the environmental variables measured in soils samples collected along an urbanization gradient in Xiamen City, China. Only the first 1st and 2nd components which collectively explained 89.25% of the total variation were displayed. The 36 sampling sites were displayed according to their corresponding ecosystem types. Prior to ordination in PCA, the environmental variables were log(x+1)-transformed, with the exception of the pH. The function ‘autoplot’ of the package *ggfortify* was used to perform PCA.


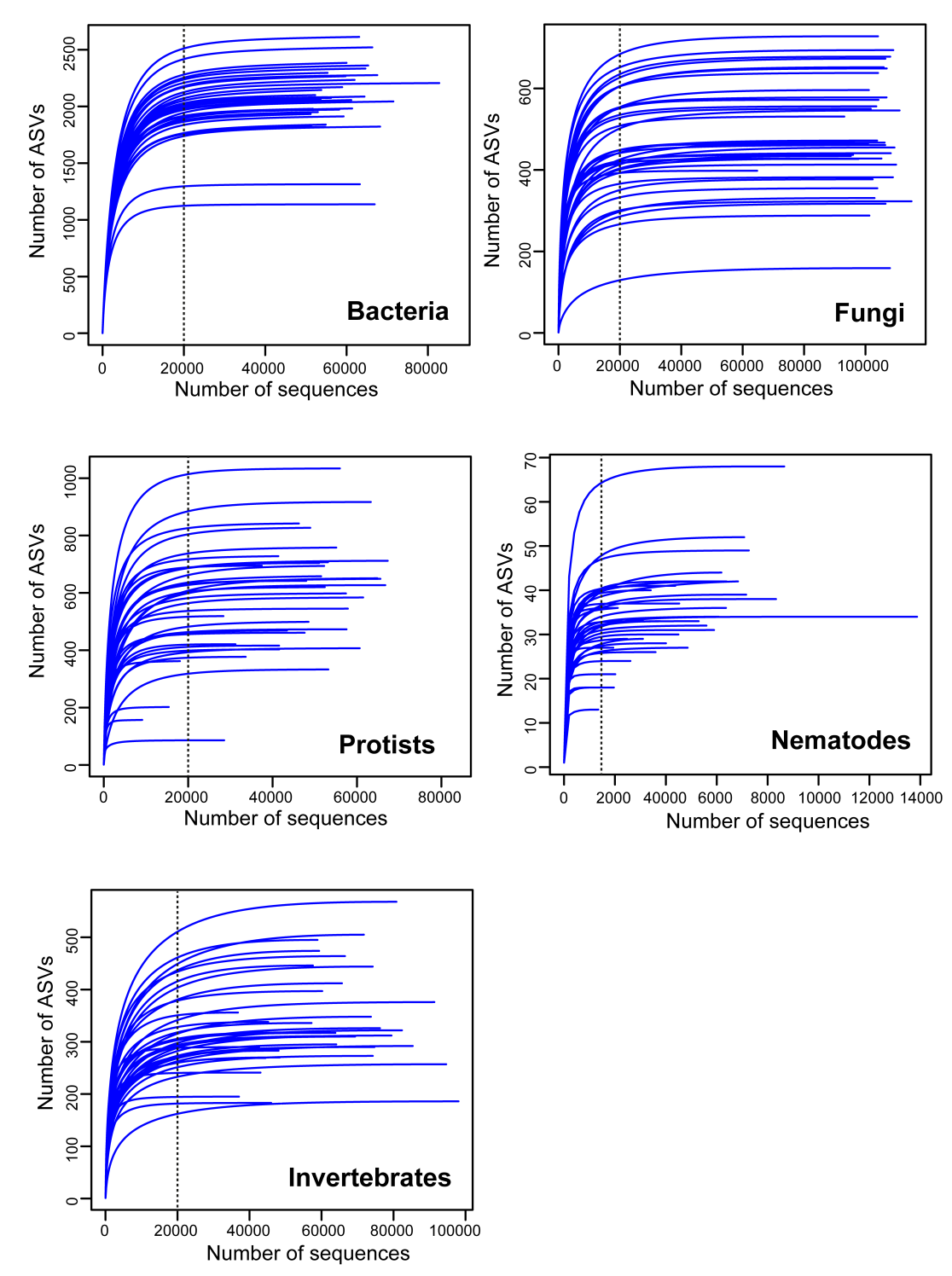


**Fig. S4** Rarefaction curves showing observed number of ASVs in soil samples collected from the 36 sites along an urbanization gradient in Xiamen City, China. The rarefaction cut-off (dotted vertical line) was set when the curves tended to plateau as only the rarest taxa remained to be sampled.


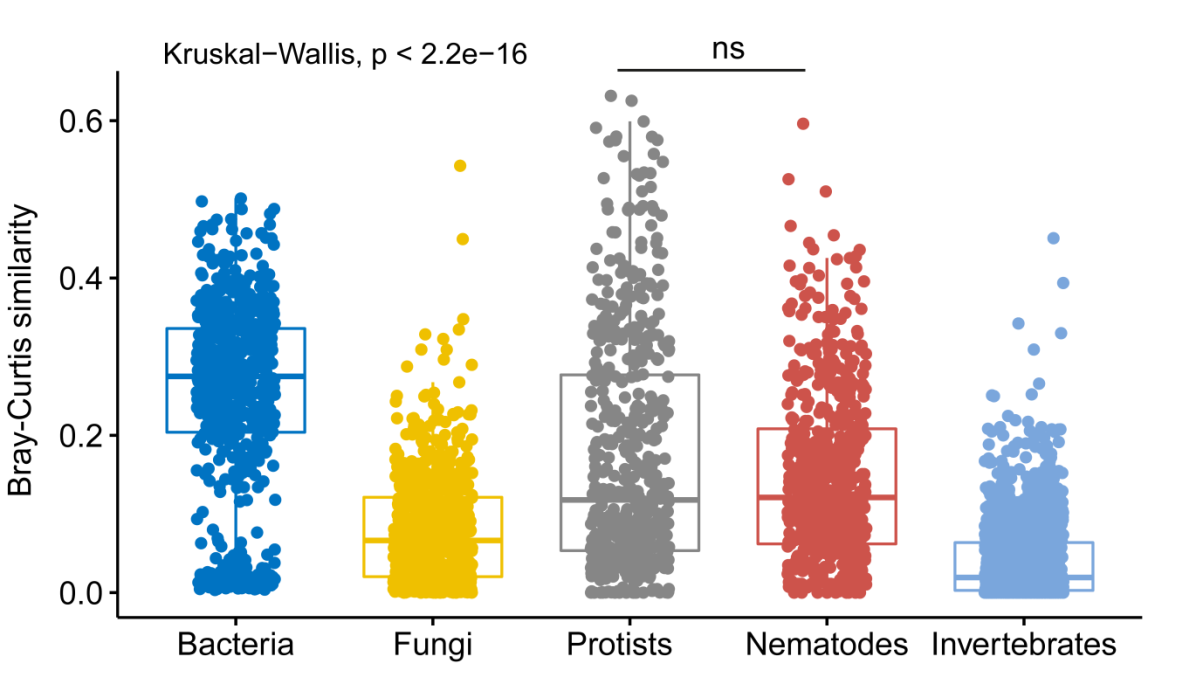


**Fig. S5** Comparison of the community similarity among different taxonomic groups of soil organisms. Grouped box plot showing pairwise Bray-Curtis similarity of each ASV (y axis) obtained during the testing phase across eight holdout subjects for each tissue type (color) and groups of soil organisms (x axis groups). All groups were significantly different from each other (significance level by Kruskal-Wallis with Dunn’s multiple comparisons test) except protists and nematodes (ns; *p* < 0.05).

**
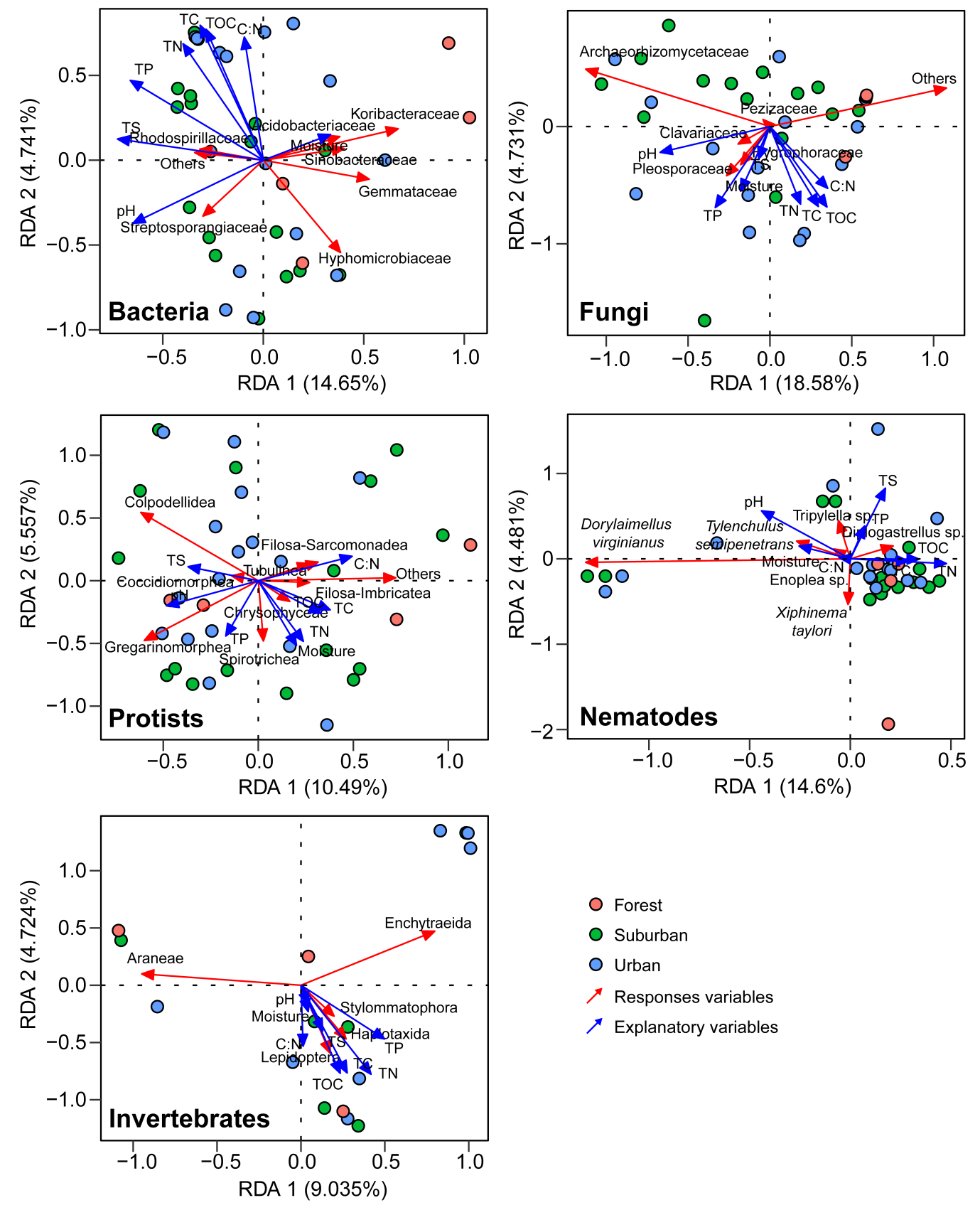
**

**Fig. S6** Redundancy analysis (RDA) plots showing the relationship between environmental variables and the locally-adapted taxa in the study area.

**R scripts used in the analyses**

**1. Sampling map**

library(raster)

setwd("C:/Users/ISABWE/Desktop/map")

ma.s <- getData("GADM", country="CHN", level=0)

ma.dem1 <- getData("SRTM", lon=117, lat=25)

plot(ma.dem1)

mid.dem <- crop(ma.dem1, extent(117.8, 118.4, 24.3, 24.8))

plot(mid.dem)

# importing sampling sites coordinates

poi <- read.csv("poi.csv")# an csv file containing geographical coordinates of the sampling sites

points(poi$lon, poi$lat)

text(poi$lon, poi$lat, labels=poi$name, poi$lat, cex=1, pos=2)

grid(col="grey50", lwd=2)

scalebar(10, below="km",type="bar", divs=4, xy=c(118.25,24.35), lonlat=TRUE, cex=1)

**2. Rarefaction**

library(phyloseq)

#Load raw ASV data of each taxonomic group (e.g., bacteria)

data<- read.table("bact.txt", row.names=1,header=T)

data= otu_table(data, taxa_are_rows = TRUE)

rarefied_data<-rarefy_even_depth(data, sample.size = 20000,

rngseed = FALSE, replace = TRUE, trimOTUs = TRUE, verbose = TRUE)

#Saving the rarefied table in the working directory

write.csv(rarefied_data, "bact_rarefied.csv")

**3. Distance-decay relationships**

library(geosphere); library(vegan); library(ecodist)

#load rarefied ASV table (e.g., bacteria)

bact<- read.table("bact_rarefied.txt", row.names=1,header=T)

bact<-t(bact)

#similarity matrix

bact_vec<-as.vector(1-(vegdist(bact, method = "bray")))

#Spatial matrix

#load geographical coordinates data

spa<- read.table("xy.txt", row.names=1,header=T)

aa<-distm(spa)

spa_vec<-as.vector(as.dist(aa)/1000)

#save both matrices

bact_spa_vec<-data.frame(spa_vec,bact_vec)

write.csv(bact_spa_vec, "bact_ddr.csv")

#Mantel test

bact_mantel<-mantel(bact_vec ~spa_vec)

bact_mantel

**4. Neutral community model**

The script for the neutral community model is available at Github at <https://github.com/Weidong-Chen-Microbial-Ecology/Stochastic-assembly-of-river-microeukaryotes> (Chen et al. 2019).

**5. Null deviation**

The script for the null deviation analysis was retrieved from Ecological Archives C002-002-S1 (Chase et al. 2011).This model expects a species by site matrix for spXsite, with row names for plots, or optionally plots named in column 1. By default calculates a modification of the Raup-Crick metric (standardizing the metric to range from -1 to 1). The above ecological archives contains a function ‘raup_crick()’ which is used for null deviation analysis.

#Null deviation of communities for bacteria

#Load the function from Ecological Archives C002-002-S1 (Chase et al. 2011)

bact=read.table("bact_rarefied.txt",row.names=1,header=T)

bact=t(bact)

bact_rc<-raup_crick(bact)

bact_rc_vec<-as.vector(bact_rc)

#save the results in the working directory

write.csv(bact_rc_vec, "bact.csv")

**6. Linear regression of community and environment**

#Environmental Euclidean distance

Env<- read.table("Env_log.txt", row.names=1,header=T)

Env_vec<-as.vector(dist(Env, method = "euclidean"))

#Combine vectors into one dataframe

all_data<-data.frame(bact_vec,fungi_vec,prot_vec,nemat_vec,invert_vec,Env_vec)

write.csv(all_data, "all_data_vect_log.csv")

#Re-arrange and re-load data for the plot

data<- read.table("all_data_vect_log.txt", row.names=1,header=T)#re-arranged data in the directory

head(data)

plot_env <- ggscatter(data, x = "Environment", y = "Dissimilarity",

add = "reg.line", # Add regression line

conf.int = TRUE, # Add confidence interval

color = "Category", palette = "jco",

point = FALSE)+stat_cor(aes(color = Category), label.x= 4)

plot_env

**7. Niche breadth analysis**

library(EcolUtils); library(spaa)

#Niche breadth of bacterial communities

bact<- read.table("bact_rarefied.txt", row.names=1,header=T)

comm.tab.bact<-t(bact)

comm.tab.bact<-comm.tab.bact[,which(colSums(comm.tab.bact)>0)]

res_bact<-spec.gen(comm.tab.bact,n=100)

#save the results in the working directory

write.csv(res_bact, "results_bact.csv")

**8. Boxplots and comparison of environmental variables**

library(ggpubr)

env=read.table("Env.txt",row.names=1,header=T)

head(env)

#example of comparison of TN data

compare_means(TN ~ Urb , data = env)

my_comparisons <- list( c("Forest", "Suburban"), c("Forest", "Urban"), c("Suburban", "Urban") )

p<-ggboxplot(env, x = "Urb", y = "TN",

color = "Urb", palette = "jco")+ stat_compare_means(label.y = 0.05)

p

**9. PCA of environmental variables**

library(ggfortify)

#load raw environnemental variables

env=read.table("Env.txt",row.names=1,header=T)

env.log<-log1p(env) #log (x+1)-transformation environnemental variables

#save transformed data

write.csv(env.log, 'env.log.csv')

#re-load after adding habitats in a column named ‘Urb’ and raw pH data

env.log=read.table("Env_log.txt",row.names=1,header=T)

head(env.log)

df <- env.log[-9]

autoplot(prcomp(df), data = env.log, colour = 'Urb',

loadings = TRUE, loadings.colour = 'blue',

loadings.label = TRUE, loadings.label.size = 5)+theme_bw()

**10. Community and environmental variables relationship**

library(vegan); library(ggcor); library(ggplot2)

#load all communities data combined

otu<- read.table("All.txt", row.names=1,header=T)

otu<-t(otu)

ncol(otu)

env <- read.csv(file = 'C:/Users/ISABWE/Desktop/06 Env_community correlation/Env.csv', row.names=1, header = TRUE)

mantel02 <- fortify_mantel(otu, env,

spec.select = list(Bacteria = 1:21842, Fungi = 21843:29519, Protists = 29520:36758,

Nematodes = 36759:37220, Invertebrates = 37221:39005)) %>%

mutate(r = cut(r, breaks = c(-Inf, 0.1, 0.25, Inf),

labels = c("<0.25", "0.25-0.5", ">=0.5"),

right = FALSE),

p.value = cut(p.value, breaks = c(-Inf, 0.001, 0.05, Inf),

labels = c("<0.05", "0.001-0.05", ">=0.05"),

right = FALSE))

quickcor(env, type = "upper") + geom_square() +

add_link(mantel02, mapping = aes(colour = p.value, size = r),

diag.label = TRUE) +

scale_size_manual(values = c(0.5, 1.5, 3)) +

geom_diag_label() + remove_axis("x")

**11. RDA of the locally-adapted taxa**

library(vegan); library(ggplot2); library(ggrepel)

fc=read.csv("C:\\Users\\ISABWE\\Desktop\\rda\\env.log.csv",header=T,row.names=1)#Reading environmental variables (explanatory data)

sp=read.csv("C:\\Users\\ISABWE\\Desktop\\rda\\bact_locally.csv",header=T,row.names =1)#Read community data as response variable data

spp=decostand(sp,method = "hellinger")#Convert response variables

fcc=log10(fc)#Converting explanatory variables

uu=rda(spp~.,fcc)#RDA Analysis

ii=summary(uu) #View analysis results

sp=as.data.frame(ii$species[,1:2])*2#Depending on the drawing result, the drawing data can be enlarged or reduced to a certain extent, as follows

st=as.data.frame(ii$sites[,1:2])

yz=as.data.frame(ii$biplot[,1:2])

grp<- read.table("group.txt", row.names=1,header=T)#group table

ggplot() +

#geom_text_repel(data = st,aes(RDA1,RDA2,label=row.names(st)),size=4)+#Show a Square

geom_point(data = st,aes(RDA1,RDA2,shape=grp$Group,fill=grp$Group),size=4)+

scale_shape_manual(values = c(21,21,21))+

geom_segment(data = sp,aes(x = 0, y = 0, xend = RDA1, yend = RDA2),

arrow = arrow(angle=22.5,length = unit(0.35,"cm"),

type = "closed"),linetype=1, size=0.6,colour = "red")+

geom_text_repel(data = sp,aes(RDA1,RDA2,label=row.names(sp)))+

geom_segment(data = yz,aes(x = 0, y = 0, xend = RDA1, yend = RDA2),

arrow = arrow(angle=22.5,length = unit(0.35,"cm"),

type = "closed"),linetype=1, size=0.6,colour = "blue")+

geom_text_repel(data = yz,aes(RDA1,RDA2,label=row.names(yz)))+

labs(x=paste("RDA 1 (", format(100 *ii$cont[[1]][2,1], digits=4), "%)", sep=""),

y=paste("RDA 2 (", format(100 *ii$cont[[1]][2,2], digits=4), "%)", sep=""))+

geom_hline(yintercept=0,linetype=3,size=1) +

geom_vline(xintercept=0,linetype=3,size=1)+

guides(shape=guide_legend(title=NULL,color="black"),

fill=guide_legend(title=NULL))+

theme_bw()+theme(panel.grid=element_blank())
